# Single-Lead ECG Recordings Including Einthoven and Wilson Leads by a Smartwatch: A New Era of Patient Directed Early ECG Differential Diagnosis of Cardiac Diseases?

**DOI:** 10.3390/s19204377

**Published:** 2019-10-10

**Authors:** Alexander Samol, Kristina Bischof, Blerim Luani, Dan Pascut, Marcus Wiemer, Sven Kaese

**Affiliations:** Department of Cardiology and Critical Care Medicine, Johannes Wesling University Hospital, Ruhr University Bochum, 32429 Minden, Germany; Kristina.Bischof@muehlenkreiskliniken.de (K.B.); Blerim.Luani@muehlenkreiskliniken.de (B.L.); DanLiviu.Pascut@muehlenkreiskliniken.de (D.P.); Marcus.Wiemer@muehlenkreiskliniken.de (M.W.); Sven.Kaese@muehlenkreiskliniken.de (S.K.)

**Keywords:** electrocardiogram (ECG), smartwatch, Apple Watch, Einthoven, Wilson, six-lead ECG, multichannel ECG

## Abstract

Background: Smartwatches that are able to record a bipolar ECG and Einthoven leads were recently described. Nevertheless, for detection of ischemia or other cardiac diseases more leads are required, especially Wilson’s chest leads. Objectives: Feasibility study of six single-lead smartwatch (Apple Watch Series 4) ECG recordings including Einthoven (I, II, III) and Wilson-like pseudo-unipolar chest leads (Wr, Wm, Wl). Methods: In 50 healthy subjects (16 males; age: 36 ± 11 years, mean ± SD) without known cardiac disorders, a standard 12-lead ECG and a six single-lead ECG using an Apple Watch Series 4 were performed under resting conditions. Recording of Einthoven I was performed with the watch on the left wrist and the right index finger on the crown, Einthoven II was recorded with the watch on the left lower abdomen and the right index finger on the crown, Einthoven III was recorded with the watch on the left lower abdomen and the left index finger on the crown. Wilson-like chest leads were recorded corresponding to the locations of V1 (Wr), V4 (Wm) and V6 (Wl) in the standard 12-lead ECG. Wr was recorded in the fourth intercostal space right parasternal, Wm was recorded in the fifth intercostal space on the midclavicular line, and Wl was recorded in the fifth intercostal space in left midaxillary line. For all Wilson-like chest lead recordings, the smartwatch was placed on the described three locations on the chest, the right index finger was placed on the crown and the left hand encompassed the right wrist. Both hands and forearms also had contact to the chest. Three experienced cardiologists were independently asked to allocate three bipolar limb smartwatch ECGs to Einthoven I–III leads, and three smartwatch Wilson-like chest ECGs (Wr, Wm, Wl) to V1, V4 and V6 in the standard 12-lead ECG for each subject. Results: All 300 smartwatch ECGs showed a signal quality useable for diagnostics with 281 ECGs of good signal quality (143 limb lead ECGs (95%), 138 chest lead ECGs (92%). Nineteen ECGs had a moderate signal quality (7 limb lead ECGs (5%), 12 chest lead ECGs (8%)). One-hundred percent of all Einthoven and 92% of all Wilson-like smartwatch ECGs were allocated correctly to corresponding leads from 12-lead ECG. Forty-six subjects (92%) were assigned correctly by all cardiologists. Allocation errors were due to similar morphologies and amplitudes in at least two of the three recorded Wilson-like leads. Despite recording with a bipolar smartwatch device, morphology of all six leads was identical to standard 12-lead ECG. In two patients with acute anterior myocardial infarction, all three cardiologists recognized the ST-elevations in Wilson-like leads and assumed an occluded left anterior descending coronary artery correctly. Conclusion: Consecutive recording of six single-lead ECGs including Einthoven and Wilson-like leads by a smartwatch is feasible with good ECG signal quality. Thus, this simulated six-lead smartwatch ECG may be useable for the detection of cardiac diseases necessitating more than one ECG lead like myocardial ischemia or more complex cardia arrhythmias.

## 1. Introduction

Smartwatches and smartphones are increasingly used worldwide. In the US, around 77% and 13% of the citizens own a smartphone and/or a smartwatch, respectively [1]. Several functions of these smart devices or apps allow the user to measure or monitor different health-related parameters like activity, energy consumption or heart rate. Some of these smart devices are approved by the FDA (US Food and Drug Administration), like the electrocardiography and photoplethysmography analysis software of the Apple Watch (Apple Inc., Cupertino, CA, USA) [2,3]. The generations of the Apple Watch Series 3^®^ applied photoplethysmography with LED lights and light-sensitive photodiodes, located on the backside of the watch, for recording of pulse frequency and irregularities [1]. A study demonstrated that detection of arrhythmias is feasible with high sensitivity and specificity [4]. A disadvantage of photoplethysmography is that this technique can only give indirect hints to the underlying rhythm, as no “real” ECG is recorded. Therefore, portable electrocardiogram (ECG) devices or handheld electrocardiogram devices like the AliveCor (AliveCor Inc., Mountain View, CA, USA) or MyDiagnostick (Applied Biomedical Systems BV, Maastricht, the Netherlands) for smartphones can record a “real” ECG [5,6,7,8]. This patient-activated recording of ECGs requires that the additional ECG device is carried along with the user. 

The Apple Watch Series 4^®^ (Apple Inc., Cupertino, CA, USA) has an integrated ECG tool which allows recording of a single-lead ECG [9,10,11]. The electrodes are placed in the crown (negative electrode) and on the back of the watch (positive electrode). A bipolar ECG-lead is derived by recording the voltage difference over time between the right index finger on the crown and the watch’s back electrode on the left arm wrist, thereby simulating Einthoven’s ECG lead I. The patient activates ECG recording and a pdf document of the ECG is generated by the Apple Health App which can be printed or sent to the doctor. This one-lead ECG can improve arrhythmia diagnosis, but discriminating P-waves are only one lead and may be challenging and insufficient for correct diagnosis of sinus rhythm [6,12]. Further, for diagnosis of sinus rhythm or other cardiac disorders, like myocardial infarction or ischemia, additional ECG leads are required. Therefore, our group recently performed a proof of concept and prospective study in healthy voluntary subjects concerning the feasibility and accuracy of recording of three consecutive single channel ECG encompassing Einthoven’s leads I, II and III by a common Apple Watch Series 4 in comparison to the same leads recorded by a standard 12-lead ECG device [10]. To the best of our knowledge this was the first study using a smartwatch for the recording of Einthoven leads I–III [10]. We demonstrated, that every subject could perform ECG recording with the smartwatch after a short introduction on how to use it [10]. ECG signal quality concerning P-wave, QRS-complex, T-wave and isoelectric line recorded by the Apple Watch seems to be highly comparable to the equivalent recorded by a commercial ECG device [10]. Recording of these additional leads by the smartwatch may improve identification of the electrical heart axis, determination of heart rhythm and detection of myocardial ischemia in the inferior myocardial area [10,13]. A case report of two patients demonstrated recording of Einthoven leads I–III by an Apple Watch in ST-elevation myocardial infarction (STEMI) [11]. The recorded ST-elevation from the Apple Watch ECG matched the waveform recorded by the standard ECG [11]. Up to now, recording Einthoven leads I–III using an Apple Watch does not cover all myocardial regions. In particular, precordial leads are required for better detection of myocardial ischemia.

The handheld electrocardiogram devices AliveCor (AliveCor Inc., Mountain View, CA, USA) and MyDiagnostick (Applied Biomedical Systems BV, Maastricht, the Netherlands) are available for smartphones and enable recording of Einthoven lead I ECG only [6,12]. The AliveCor device has a high sensitivity and specificity for Atrial fibrillation (AF) detection but the study also showed false positive AF detection due to small voltage P waves in lead I [12]. Another study evaluated the two handheld ECG devices for AF screening in a hospital population in cardiologic and geriatric patients and found that sensitivity and specificity of the automated algorithms were suboptimal, requiring additional standard 12-lead ECGs for optimizing specificity [6]. 

Most handheld ECG devices are limited as only one ECG lead, Einthoven I, can be acquired. Due to this, diagnosing cardiac arrhythmias and repolarization disorders is hampered. Further, users must carry the handheld device in addition to their smart phone, which may be inconvenient and impractical in daily life. 

Our group recently demonstrated that patient-directed ECG recording of Einthoven leads I–III can easily be performed by using an Apple Watch [10]. Until now, a limitation of the Apple Watch is that precordial ECG leads can not be recorded, which would be necessary e.g., for detection of myocardial ischemia in other regions of the heart as recorded in Einthoven leads I–III. To the best of our knowledge, this is the first study using an Apple Watch Series 4 for recording of Wilson-like precordial ECG leads. We recorded three Wilson-like smart watch ECGs (Wr, Wm, Wl) corresponding to Wilson leads V1, V4 and V6 of the standard 12-lead ECG. Recording of only three Wilson-like ECGs was performed to keep ECG recording as simple as possible for the patients. In addition, we recorded Einthoven I–III in each patient, thus generating a six-lead smartwatch ECG.

## 2. Materials and Methods

### 2.1. Study Participants

This study was approved by the local Ethics Committee of the Aerztekammer Westfalen-Lippe (reference number 2019-456) and performed in accordance to the Declaration of Helsinki. Fifty healthy voluntary subjects (16 males; mean age 36 ± 11 years) with no history of cardiovascular disease were prospectively enrolled in our study.

Additionally, we performed smartwatch ECG measurements in two male patients with acute anterior myocardial infarction to demonstrate the feasibility of ischemia detection by smartwatch ECG recording. Both patients went to the emergency room on their own with acute chest pain. Smartwatch ECGs were recorded during preparation of the catheter lab for percutaneous coronary intervention.

### 2.2. 12-Lead ECG

Standard 12-lead ECGs were recorded using a common ECG device (MAC 5500, GE Healthcare, Chicago, IL, USA) with a paper running speed of 50 mm/s. All ECG recordings were performed after a resting period of 5 min in supine position.

### 2.3. Smartwatch ECG Recordings

**Figure 1:**
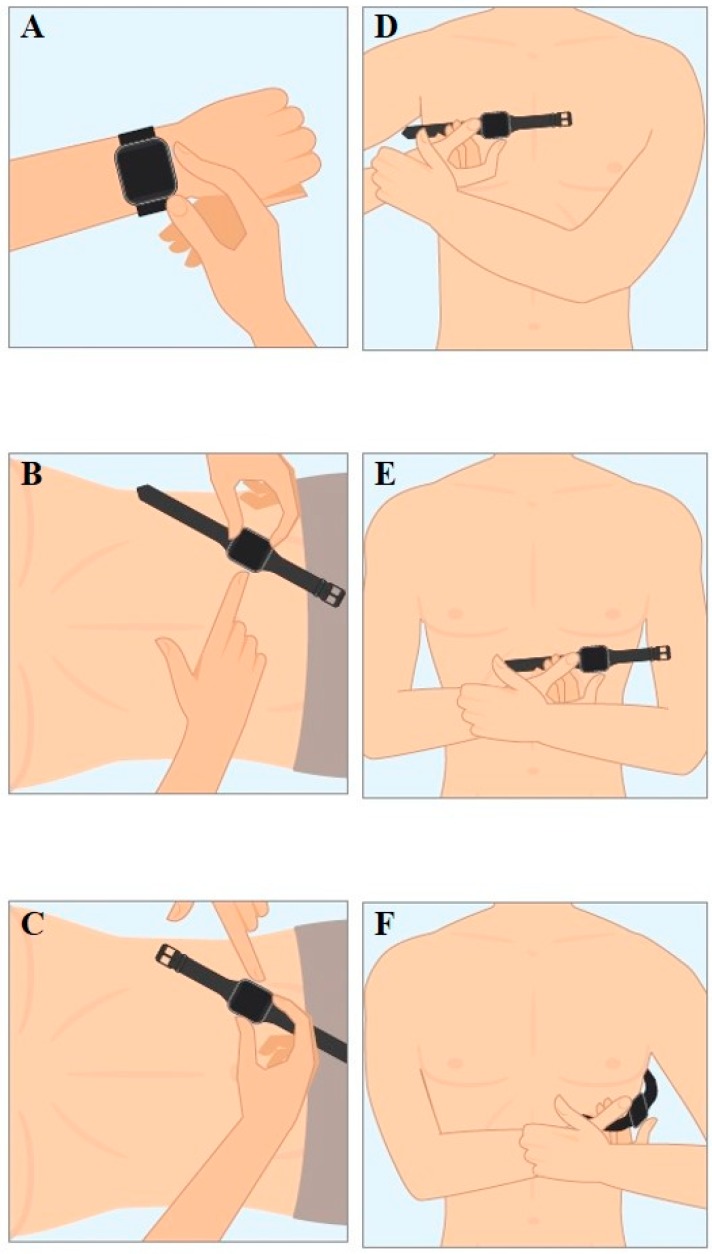
(**A**) Recording of Einthoven lead I between the left arm wrist and the right index finger. (**B**) Recording of Einthoven lead II between the left lower abdominal region and the right index finger. (**C**) Recording of Einthoven lead III between the left lower abdominal region and the left index finger. (**D**) Recording of Wilson-like right (Wr) with smartwatch at the fourth intercostal space right parasternal. (**E**) Recording of Wilson-like medial (Wm) with the smartwatch at the fifth intercostal space midclavicular line. (**F**) Recording of Wilson-like left (Wl) with the smartwatch at the fifth intercostal space in left midaxillary line.

An Apple Watch Series 4^®^ (Apple Inc., Cupertino, CA, USA) was used for the six single-lead ECG recordings immediately after recording the 12-lead ECG. Recording procedure of Einthoven leads I–III with the Apple Watch has been described earlier [10]. In brief, Einthoven I was recorded with the Apple Watch on the left wrist and the right index finger on the crown (Figure 1A), recording of Einthoven II was performed with the watch on the left lower abdomen and the right index finger on the crown (Figure 1B), and Einthoven III with the watch on the left lower abdomen and the left index finger on the crown (Figure 1C). The pseudo-unipolar Wilson-like chest leads were recorded corresponding to the locations of V1, V4 and V6 in the standard 12-lead ECG. Wilson-like right (Wr) (Figure 1D) corresponded to V1 and was recorded with the smartwatch placed at the fourth intercostal space right parasternal. Wilson-like medial (Wm) (Figure 1E) corresponded to V4 with the smartwatch placed at the fifth intercostal space on the midclavicular line. Wilson-like left (Wl) corresponded to V6 with the smartwatch at the fifth intercostal space in left midaxillary line (Figure 1F). For all Wilson-like chest lead recordings, the smartwatch was placed on the described three locations on the chest, the right index finger was placed on the crown and the left hand encompassed the right wrist. Both hands and forearms also had contact to the chest. 

All recorded ECGs were digitally stored using the Health Application of an iPhone Series XR (Apple Inc., Cupertino, CA, USA).

All single-lead smartwatch ECGs were automatically converted to a pdf document using the “send pdf to your doctor” function and the paper was printed for further analysis. All recorded ECGs were classified to be of moderate signal quality if at least three consecutive QRS-complexes showed noise-free signal quality inclusive of no artifacts in iso-electric lines between QRS-complexes. ECGs were classified to be of good signal quality if at least ten QRS-complexes showed noise-free signal quality and no artifacts in iso-electric lines between QRS-complexes.

Three experienced cardiologists were independently asked to assign the three limb lead Apple Watch ECG recordings to Einthoven I–III leads from standard 12-lead ECGs, and the three Wilson-like chest lead Apple Watch ECGs to V1, V4 and V6 from standard 12-lead ECG for each subject. In order to be blinded, the six Apple Watch ECG paper prints were randomly labeled “EI”, “EII”, “EIII”, “WI”, “WII” or “WIII” for each subject. The cardiologist assigned each single Apple Watch ECG by visual comparison to Einthoven’s leads I–III and Wilson standard chest leads V1, V4 and V6 from 12-lead ECG. Thereafter, two physicians not involved in the comparison used a prepared solution table to check if the assignment was correct.

### 2.4. Statistical Analysis

Statistical analysis was performed using IBM SPSS Statistics (version 24 for Mac, IBM Corporation, Somers, NY, USA). Categorical variables are shown as absolute numbers and percentages. Continuous variables are presented as mean ± standard deviation. For assessment of differences of metric outcome variables, we used one-way repeated analysis of variance (ANOVA) and paired *T*-Test. In case of binary variables, we used the χ2-test.

## 3. Results

**Figure 2:**
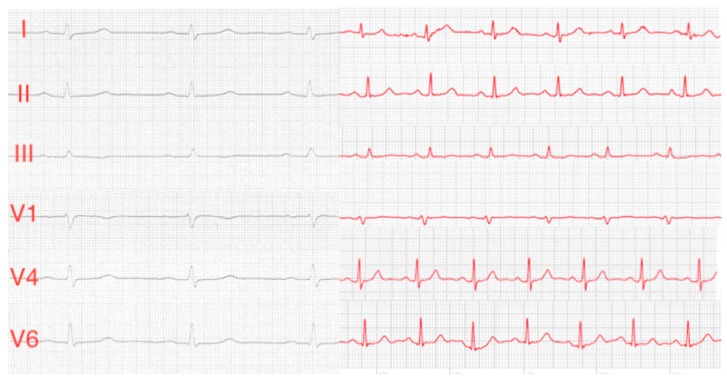
Comparison of typical standard Einthoven I–III leads and Wilson chest leads V1, V4 and V6 from standard ECG (black ECG curves) and Apple Watch ECGs (red ECG curves).

Subject characteristics are shown in Table 1. After a short instruction, all 50 healthy subjects were able to perform the ECG recordings with the Apple Watch on their own. All 300 Apple Watch ECGs showed an adequate signal quality for diagnostics purposes with 143 limb lead ECGs (95%) and 138 chest lead ECGs (92%) of good signal quality and 7 limb lead ECGs (5%) and 12 chest lead ECGs (8%) of moderate signal quality. Thus, 281 of all 300 ECGs showed good signal quality with only 19 ECGs of moderate signal quality. One hundred percent of all Apple Watch Einthoven ECGs, and ninety-two percent of all Apple Watch Wilson-like ECG recordings were correctly assigned to the corresponding leads of standard 12-lead ECG by the three cardiologists. Correct assignment of all six single-lead ECGs to the corresponding leads ranged from 47 to 49 (94% to 98%). Forty-six subjects (92%) were correctly assigned by all three cardiologists. Typical ECG recordings are shown in Figure 2. In one of the three subjects with at least one incorrect assignment, all cardiologists allocated the Wilson-like ECGs incorrectly. All assignment errors were made in subjects with comparable morphologies and amplitudes in at least two of the three Wilson-like leads recorded by the Apple Watch and/or the standard ECG device. In all subjects with incorrect assignment of Wilson-like leads at least one Wilson-like ECG lead was assigned correctly. In the one subject with assignment errors by all cardiologists, the correctly identified ECG lead was the same throughout the results of all three cardiologists. We also performed Fleiss’ kappa analysis which showed moderate interrater reliability (kappa 0.479, *P* < 0.001) and calculated an intraclass correlation coefficient of 0.739. Subjects with assignment errors showed no significant differences in subjects’ age, weight, height, body mass index (BMI), body surface area (BSA), sex, heart rate and electrical heart axis (Table 2).

**Figure 3:**
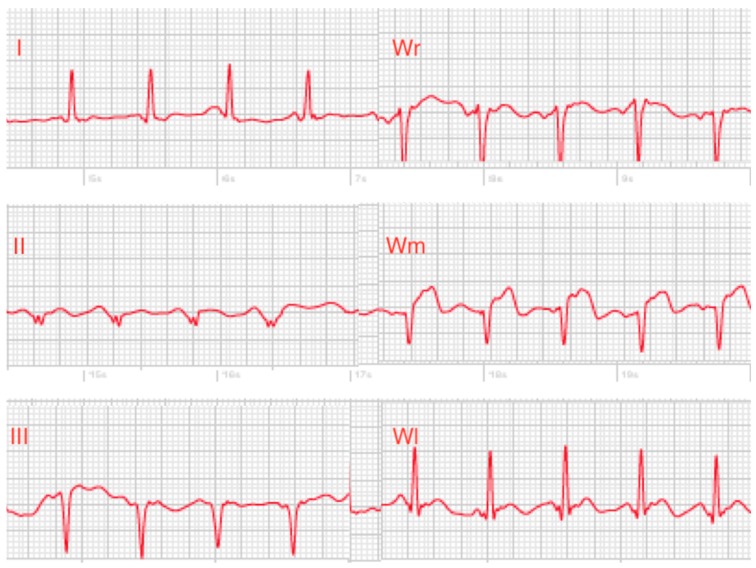
Smartwatch ECGs of a patient with acute anterior myocardial infarction with clear ST-elevations in lead Wm. (I = Einthoven lead I, II = Einthoven lead II, III = Einthoven lead III, Wr = pseudo-unipolar recording corresponding to standard lead V1, Wm = pseudo-unipolar recording corresponding to standard lead V4, Wl = pseudo-unipolar recording corresponding to standard lead V6).

In two male patients with acute anterior myocardial infarction, all three cardiologists were able to clearly recognize ST-elevations in the smartwatch chest lead recordings and allocated the assumed occluded vessel correctly to left anterior descending artery. Signal quality of the recorded single-lead ECGs was moderate (Figure 3).

## 4. Discussion

Smart devices like smartphones and smartwatches are used worldwide and have become an integral part of daily life [1]. These smart devices and apps are also increasingly used in medical care and therefore, studies need to be performed in order to evaluate their reliability and accuracy concerning the effective use for promotion of health [9]. In this context, the FDA has approved the electrocardiography and photoplethysmography analysis software of the Apple Watch (Apple Inc., Cupertino, CA, USA) [2,3]. 

To the best of our knowledge, this is the first study which evaluated the feasibility of an Apple Watch Series 4 to record three Wilson-like precordial ECG leads corresponding to the standard Wilson leads V1, V4 and V6 of a standard 12-lead ECG. Together with the recently reported recording of Einthoven leads I–III using an Apple Watch [10], we demonstrated the use of an Apple Watch for recording a six consecutive single-lead smartwatch ECG, simulating a 6-channel ECG.

After a short introduction, each of the 50 healthy subjects could perform ECG recordings with the Apple Watch by themselves in the six required positions for recording Einthoven leads I–III and Wilson-like (Wr, Wm, Wl) ECGs. These results are in accordance with our previous study in which we only recorded Einthoven leads I–III using the Apple smartwatch [10]. Our findings may indicate that appropriate use of the smartwatch for six single-lead ECG recordings is feasible after a short instruction and therefore may be widely used in the general population. Our study cohort consisted of healthy middle-aged subjects who are more familiar with smart devices compared to older subjects. Therefore, adequate application of a smartwatch for ECG recordings in different positions by older subjects might be less practicable. This assumption may be supported by a study which showed that 7% of cardiology and 21.4% of geriatric patients could not appropriately use handheld electrocardiogram devices for single-lead ECG recordings [6]. 

Signal quality of all 300 Apple Watch ECGs was sufficient for accurate diagnostic evaluation. In our previous study, 91% of all Apple Watch ECGs could correctly be assigned to Einthoven leads I, II and III recorded by the standard ECG [10]. These results were confirmed by our current study, which demonstrated that now 100 percent of all Apple Watch Einthoven ECGs were allocated correctly to the standard ECG. Further, these findings may point to an improved learning curve of recording Einthoven leads I–III ECGs by an Apple Watch. 

Our study showed that assignment errors were made in subjects with comparable morphologies and amplitudes in at least two of the three Wilson-like leads recorded by the smartwatch and/or the standard ECG device. Assignment errors were not related to significant differences in subjects’ age, weight, height, BMI, BSA, sex, heart rate and electrical heart axis (Table 2). These data may indicate that sufficient smartwatch ECG recording with good signal quality seems to be feasible and independent of patients’ characteristics, such as physical build. 

The most common cardiac arrhythmia is AF, which is responsible for up to 25% of strokes in the US [1]. Unfortunately, in 18% of AF-associated stroke, AF is detected only after the stroke event [1]. In recent studies, smartwatches were primarily used for evaluation of their capabilities to detect AF [1,4]. The Apple Heart Study used photoplethysmography of an Apple Watch for identification of pulse irregularity, which may indicate AF [1]. The authors showed that the Apple Watch could reliably detect pulse irregularity which might unmask asymptomatic AF [1,12]. The WATCH AF study also applied photoplethysmography and a smartwatch-based algorithm in comparison with a standard ECG for AF detection [4]. The study demonstrated feasibility and very high diagnostic accuracy of AF detection by a smartwatch [4]. Of note, the study had a high dropout rated due to insufficient signal quality of the smartwatch-based algorithm [4]. In accordance with the two aforementioned smartwatch studies [1,4], another study also showed that smartwatch photoplethysmography was capable of detecting AF but also described reduced sensitivity and specificity compared to the standard ECG [14].

A major limitation of photoplethysmography is that the technique can only detect the patients pulse and recorded pulse irregularities are used as a surrogate for the detection of AF [1,4,14]. Pulse irregularities may also underlie supraventricular and ventricular extra systoles which may be misinterpreted as AF by photoplethysmography. Due to this limitation of photoplethysmography, recording of a smartwatch ECG with detection of P waves may increase accuracy in arrhythmia detection. 

Different handheld electrocardiogram devices as an add-on tool for e.g., smartphones are available which allow recording of a single-lead ECG. The AliveCor device can record Einthoven lead I and was used for community AF screening [12]. The study showed a sensitivity of 98% and specificity of 97% for AF detection [12]. However, small voltage of P waves in lead I was responsible for false positive AF detection [12]. In addition, another study evaluated single-lead ECG devices for AF screening in cardiologic and geriatric in-hospital patients [6]. The used automated algorithms had suboptimal sensitivity and specificity (cardiology: 81.8 and 94.2%, respectively, for MyDiagnostick; 54.5 and 97.5%, respectively, for AliveCor; Geriatrics: 89.5 and 95.7%, respectively, for MyDiagnostick; 78.9 and 97.9%, respectively, for AliveCor), requiring additional standard 12-lead ECG recordings to improve specificity [6]. In accordance, Lau et. al used a single-lead ECG device for recording of Einthoven lead I and demonstrated a very high sensitivity of 95–100% and specificity of 90–97% for AF detection [12]. This study also showed that false positive AF detection was due to small voltage P waves in lead I [12]. A fundamental handicap of most single-lead ECG devices is that only ECG lead Einthoven I can be recorded, which limits detection of arrhythmias or repolarization disorders. The newer generation AliveCor device has a third electrode on its backside, which allows simultaneous recording of a 6-channel bipolar ECG including all Einthoven and Goldberger leads. Nevertheless, this device is not able to record Wilson leads simultaneously with the limb leads. 

A case series of five patients demonstrated recording of a standard 12-lead ECG by the bipolar AliveCor (AliveCor Inc., Mountain View, CA, USA) handheld ECG device, which was modified with standard ECG tabs and wires with alligator clips [5]. Although ECG recording could be performed with good signal quality, the required modification of the device with additional tabs and wires is not suitable for patient-directed self ECG recording outside a clinical study [5].

Therefore, by recording six single-lead ECGs with the new generation of the Apple smartwatch, detection of cardiac arrhythmias may be more reliable than application of photoplethysmography. In summary, recordings from smartwatch ECG leads Einthoven I–III [10], as well as recordings from smartwatch Wilson-like ECGs—which together generate a six-lead smartwatch ECG—may improve ECG quality and enhance sensitivity and specificity for arrhythmia detection, e.g., for AF detection. Further, a smartwatch derived six single-lead ECG may also allow differential detection of other cardiac arrhythmias. As smartwatches are increasingly used worldwide, increasing patient-directed application of smartwatches may improve ECG detection of cardiac diseases. A simultaneous recording of multi-channel ECGs with a smartwatch is not yet possible. Our main reason for not performing recordings of all six Wilson chest leads was the complexity of this process and the time for recordings. But the main advantage of smartwatch ECG recordings is that patients wear these devices in their daily life, thus they do not have to carry along a special device for medical diagnostics.

Besides the use of smartwatches for arrhythmia detection, smartwatch ECG recording may also be used for the detection of repolarization disorders like in patients with myocardial infarction [11]. A case report of two patients demonstrated recording of Einthoven leads I–III by an Apple Watch in ST-elevation myocardial infarction [11]. The recorded ST-elevation in the smartwatch ECGs were comparable to the standard ECG [11]. Up to now, recording of Einthoven leads I–III by the Apple Watch does only partly cover regions of the heart. Especially, precordial leads are required for better detection of repolarization abnormalities or myocardial ischemia. Our study demonstrated the feasibility of Wilson-like smartwatch ECG recordings in three different positions which are comparable to Wilson V1, V4 and V6 of the standard ECG. Together with the smartwatch Einthoven leads I–III, this six single-lead smartwatch ECG covers larger areas of the myocardium, which may improve detection of repolarization disorders and myocardial ischemia. In a case series of two patients with acute anterior myocardial infarction we were able to identify ST-elevations clearly in the smartwatch chest lead ECGs. Nevertheless, the recordings were performed at a hospital with assistance, and not by the patients on their own at home.

Recording of a six single-lead ECG with an Apple Watch Series 4 is feasible with good ECG signal quality in healthy subjects. This device has the potential to play an important role in detection of arrhythmias and repolarization abnormalities, which might initiate early diagnosis of cardiac disorders by patients themselves. We demonstrated feasibility of extended ECG recording with a smartwatch as a proof of concept and demonstrated the first detection of an anterior myocardial infarction. The diagnostic potential for detection of cardiac diseases needs to be evaluated in further, larger studies.

## 5. Conclusions

Recording of consecutive six single-lead smartwatch ECGs encompassing Einthoven leads I–III and Wilson-like chest leads (Wr, Wm, Wl) with an Apple Watch Series 4 is feasible with good ECG signal quality for diagnostic purposes. The smartwatch ECG leads were highly comparable to the same leads in the standard 12-lead ECG. After subjects are provided with a short instruction, they are able to record the six-lead smartwatch ECG by themselves. Due to the increased leads, which can now be recorded by a smartwatch, this device may have an important role in patient-directed detection of cardiac arrhythmias and repolarization abnormalities, which in turn might shorten time delay to sufficient diagnosis and treatment. 

## 6. Limitations

The smartwatch ECG leads and leads of the standard ECG were only compared visually by three cardiologists. Until now, there is no computer-based comparison of the smartwatch ECGs with the 12-lead ECG available. Further, smartwatch ECGs were recorded with a paper running speed of 25 mm/s, whereas standard 12-lead ECGs were recorded with a paper running speed of 50 mm/s, which might have influenced correct smartwatch ECG allocation to the 12-lead standard ECG. Our pilot study was designed as a proof of concept. Due to this, we evaluated feasibility of smartwatch ECG recordings in healthy subjects. So far, we have only performed two measurements in patients with myocardial infarction. Now, after demonstrating feasibility of six single-lead ECG recordings in standard positions in healthy subjects and in a case series of two patients with myocardial infarction by a smartwatch, further studies are required to evaluate detection accuracy and feasibility in patients with acute myocardial infarction or arrhythmia.

## 7. Legends

Figure 1A: Recording of Einthoven lead I between the left arm wrist and the right index finger. B: Recording of Einthoven lead II between the left lower abdominal region and the right index finger. C: Recording of Einthoven lead III between the left lower abdominal region and the left index finger. D: Recording of Wilson-like right (Wr) with smartwatch at the fourth intercostal space right parasternal. E: Recording of Wilson-like medial (Wm) with the smartwatch at the fifth intercostal space midclavicular line. F: Recording of Wilson-like left (Wl) with the smartwatch at the fifth intercostal space in left midaxillary line.

Table 1. Subject characteristics (BSA = body surface area, BMI = body mass index, HR = heart rate, lead I = Einthoven lead I, lead II = Einthoven lead II, lead III = Einthoven lead III, Wilson r = pseudo-unipolar recording corresponding to standard lead V1, Wilson m = pseudo-unipolar recording corresponding to standard lead V4, Wilson l = pseudo-unipolar recording corresponding to standard lead V6). *P* values were obtained by *T*-Test and ANOVA; probability limit 95%.

Figure 2. Comparison of typical standard Einthoven I–III leads and Wilson chest leads V1, V4 and V6 from standard ECG (black ECG curves) and Apple Watch ECGs (red ECG curves).

Table 2. Correlations between correct ECG assumption and study population characteristics (BSA = body surface area, BMI = body mass index, HR = heart rate, lead I = Einthoven lead I, lead II = Einthoven lead II, lead III = Einthoven lead III, Wilson r = pseudo-unipolar recording corresponding to standard lead V1, Wilson m = pseudo-unipolar recording corresponding to standard lead V4, Wilson l = pseudo-unipolar recording corresponding to standard lead V6). P values were obtained by T-Test, ANOVA and χ2-test; probability limit 95%.

## Figures and Tables

**Table 1:** Subject characteristics (BSA = body surface area, BMI = body mass index, HR = heart rate, lead I = Einthoven lead I, lead II = Einthoven lead II, lead III = Einthoven lead III, Wr = pseudo-unipolar recording corresponding to standard lead V1, Wm = pseudo-unipolar recording corresponding to standard lead V4, Wl = pseudo-unipolar recording corresponding to standard lead V6). P values were obtained by T-Test and ANOVA; probability limit 95%.

	All	Male	Female	p
**Size (cm)**	169 ± 25	170 ± 45	169 ± 6	0.910
**Weight (kg)**	70 ± 11	78 ± 11	66 ± 9	<0.001
**BSA (m^2^)**	1.78 ± 0.29	1.86 ± 0.47	1.75 ± 0.14	0.205
**BMI (kg/m^2^)**	23.2 ± 2.7	23.8 ± 2.6	23.0 ± 2.7	0.333
**Age (years)**	36 ± 11	36 ± 10	37 ± 12	0.769
**QRS axis (°)**	54 ± 30	47 ± 31	57 ± 29	0.260
**HR 12 lead ECG (bpm)**	71 ± 13	72 ± 11	72 ± 12	0.896
**HR lead I (bpm)**	71 ± 10	71 ± 9	71 ± 11	0.900
**HR lead II (bpm)**	71 ± 10	71 ± 9	72 ± 11	0.758
**HR lead III (bpm)**	71 ± 9	72 ± 8	71 ± 10	0.830
**HR Wr (bpm)**	71 ± 10	71 ± 8	71 ± 10	0.982
**HR Wm (bpm)**	72 ± 10	72 ± 9	72 ± 11	0.993
**HR Wl (bpm)**	72 ± 10	73 ± 9	72 ± 12	0.948

**Table 2:** Correlations between correct ECG assumption and study population characteristics (BSA = body surface area, BMI = body mass index, HR = heart rate, lead I = Einthoven lead I, lead II = Einthoven lead II, lead III = Einthoven lead III, Wr = pseudo-unipolar recording corresponding to standard lead V1, Wm = pseudo-unipolar recording corresponding to standard lead V4, Wl = pseudo-unipolar recording corresponding to standard lead V6). P values were obtained by T-Test, ANOVA and χ2-test; probability limit 95%.

	ECG Correct	ECG Incorrect	p
**Size (cm)**	168 ± 26	176 ± 10	0.573
**Weight (kg)**	69 ± 10	75 ± 22	0.364
**BSA (m^2^)**	1.78 ± 0.28	1.90 ± 0.32	0.416
**BMI (kg/m^2^)**	23.2 ± 2.6	23.7 ± 4.2	0.725
**Age (years)**	37 ± 11	28 ± 7	0.112
**QRS axis (°)**	55 ± 29	43 ± 44	0.450
**Sex (m/f)**	13/26	5/6	0.459
**HR 12 lead ECG (bpm)**	71 ± 13	69 ± 11	0.511
**HR lead I (bpm)**	71 ± 10	73 ± 9	0.782
**HR lead II (bpm)**	71 ± 10	70 ± 12	0.806
**HR lead III (bpm)**	71 ± 9	71 ± 11	0.889
**HR Wr (bpm)**	71 ± 10	68 ± 10	0.550
**HR Wm (bpm)**	72 ± 10	70 ± 9	0.751
**HR Wl (bpm)**	72 ± 11	73 ± 12	0.882

## References

[1] Turakhia M.P., Desai M., Hedlin H., Rajmane A., Talati N., Ferris T., Desai S., Nag D., Patel M., Kowey P. (2019). Rationale and design of a large-scale, app-based study to identify cardiac arrhythmias using a smartwatch: The Apple Heart Study. Am. Heart J..

[2] FDA. https://www.accessdata.fda.gov/cdrh_docs/pdf18/DEN180042.pdf.

[3] https://www.accessdata.fda.gov/cdrh_docs/pdf18/DEN180044.pdf.

[4] Dorr M., Nohturfft V., Brasier N., Bosshard E., Djurdjevic A., Gross S., Raichle C.J., Rhinisperger M., Stockli R., Eckstein J. (2019). The WATCH AF Trial: SmartWATCHes for Detection of Atrial Fibrillation. JACC Clin. Electrophysiol..

[5] Baquero G.A., Banchs J.E., Ahmed S., Naccarelli G.V., Luck J.C. (2015). Surface 12 lead electrocardiogram recordings using smart phone technology. J. Electrocardiol..

[6] Desteghe L., Raymaekers Z., Lutin M., Vijgen J., Dilling-Boer D., Koopman P., Schurmans J., Vanduynhoven P., Dendale P., Heidbuchel H. (2017). Performance of handheld electrocardiogram devices to detect atrial fibrillation in a cardiology and geriatric ward setting. EP Europace.

[7] Samol A., Masin M., Gellner R., Otte B., Pavenstadt H.J., Ringelstein E.B., Reinecke H., Waltenberger J., Kirchhof P. (2013). Prevalence of unknown atrial fibrillation in patients with risk factors. EP Europace.

[8] Nigolian A., Dayal N., Nigolian H., Stettler C., Burri H. (2018). Diagnostic accuracy of multi-lead ECGs obtained using a pocket-sized bipolar handheld event recorder. J. Electrocardiol..

[9] Foster K.R., Torous J. (2019). The Opportunity and Obstacles for Smartwatches and Wearable Sensors. IEEE Pulse.

[10] Samol A., Bischof K., Luani B., Pascut D., Wiemer M., Kaese S. (2019). Recording of Bipolar Multichannel ECGs by a Smartwatch: Modern ECG Diagnostic 100 Years after Einthoven. Sensors.

[11] Avila C.O. (2019). Novel Use of Apple Watch 4 to Obtain 3-Lead Electrocardiogram and Detect Cardiac Ischemia. Perm. J..

[12] Lau J.K., Lowres N., Neubeck L., Brieger D.B., Sy R.W., Galloway C.D., Albert D.E., Freedman S.B. (2013). iPhone ECG application for community screening to detect silent atrial fibrillation: A novel technology to prevent stroke. Int. J. Cardiol..

[13] Burch G.E. (1978). History of precordial leads in electrocardiography. Eur. J. Cardiol..

[14] Tison G.H., Sanchez J.M., Ballinger B., Singh A., Olgin J.E., Pletcher M.J., Vittinghoff E., Lee E.S., Fan S.M., Gladstone R.A. (2018). Passive Detection of Atrial Fibrillation Using a Commercially Available Smartwatch. JAMA Cardiol..

